# The therapeutic promise of probiotic *Bacteroides fragilis* (BF839) in cancer immunotherapy

**DOI:** 10.3389/fmicb.2025.1523754

**Published:** 2025-03-31

**Authors:** Kunwei Peng, Yuqing Li, Qijun Yang, Peijin Yu, Ting Zeng, Chuhui Lin, Yuhong Deng, Jingqi Chen

**Affiliations:** ^1^Department of Medical Oncology, The Second Affiliated Hospital, Guangzhou Medical University, Guangzhou, China; ^2^Guangzhou Key Laboratory for Research and Development of Nano-Biomedical Technology for Diagnosis and Therapy, The Second Affiliated Hospital, Guangzhou Medical University, Guangzhou, China; ^3^Guangdong Provincial Education Department Key Laboratory of Nano-Immunoregulation Tumour Microenvironment, The Second Affiliated Hospital, Guangzhou Medical University, Guangzhou, China; ^4^Department of Clinical Nutrition, The Second Affiliated Hospital, Guangzhou Medical University, Guangzhou, China

**Keywords:** *Bacteroides fragilis*, gut microbiota, tumor microenvironment, immune checkpoint inhibitors, immunotherapy

## Abstract

**Background:**

Overwhelming evidence suggests that the gut microbiota modulates tumor response to immune checkpoint inhibitors (ICIs). The probiotic *Bacteroides fragilis* (BF839) was extensively used in China to improve gut microbiota dysbiosis-related symptoms. We hypothesized that probiotic BF839 could enhance tumor sensitivity to ICIs.

**Methods:**

In the preclinical studies, mice received BF839 orally, PD-1 intraperitoneal injection, or a combination therapy of the two agents. The antitumor effect of BF839 was investigated by assessing the tumor growth and tumor immune microenvironment. Mice fecal samples were collected for 16S rRNA sequencing. Fresh tumor samples were collected for 16S RNA sequencing. The data of 29 patients with advanced solid tumor who received BF839 adjuvant therapy were retrospectively evaluated. The primary endpoint was overall survival (OS).

**Results:**

Among patients with advanced solid tumors undergoing ICIs and chemotherapy, patients in BF839 long-term adjuvant treatment group had longer OS (*p* = 0.0101) than the BF839 short-term adjuvant treatment group. In the preclinical studies, we found that monotherapy with BF839 or anti-PD-1 antibody significantly inhibit tumor growth. Interestingly, BF839 worked synergistically with anti-PD-1 antibody and induced tumor regression, mediated by increased CD8^+^T cell infiltration. Mechanistically, BF839 induced tumor suppression was regulated by the cGAS-STING pathway. 16S rRNA sequencing results of mice fecal samples showed that BF839 treatment increased gut microbiota diversity.

**Conclusion:**

Overall, our data suggest that BF839 enhanced tumor sensitivity to ICIs through cGAS-STING signaling. In the future, the application of probiotic BF839 to regulate gut microbiota may be a new strategy to enhance the efficacy of ICIs.

## 1 Introduction

In recent years, immune checkpoint inhibitors (ICIs), especially monoclonal antibody targeting immune checkpoints such as programmed cell death protein 1 (PD-1), programmed death-ligand 1 (PD-L1) and cytotoxic T-lymphocyte-associated protein 4 (CTLA-4) have transformed the treatment of many cancers (Kennedy and Salama, 2020). ICIs have received the approval of the US Food and Drug Administration (FDA) for non-small cell lung cancer (NSCLC), hepatocellular carcinoma (HCC), nasopharyngeal cancer, triple-negative breast cancer, high microsatellite instability (MSI-H) tumors and others (Forde et al., 2022; Qin et al., 2023; Chan et al., 2023; Cortes et al., 2022; Ludford et al., 2023). It has been established that tumor immunotherapy relies mainly on stimulating or rebuilding the host immune system to suppress or kill cancer cells, and offers various advantages, such as low toxicity and high efficiency (Yang, 2015). However, it should be borne in mind that many patients do not respond to ICIs, requiring combination therapy to maximize the benefit of immunotherapy. Studies of combination therapy mainly focused on chemotherapy, antiangiogenic therapy, and targeted therapy, however, they bring more adverse events (Park et al., 2024; Mok et al., 2024; Motzer et al., 2024). Immunomodulators, including probiotic, has become a hot research topic (Wang et al., 2018).

Gut microbiota maintains a symbiotic relationship with intestinal mucosa and plays an important role in metabolism, immunity, intestinal protection and cancer screening in healthy individuals (Brezina et al., 2023; Erttmann et al., 2022; Liu et al., 2024). Regarding anti-tumor effects, the function of gut microbiota in mediating immune activation has been substantiated (Li et al., 2022; Mattiola and Diefenbach, 2023). Besides, studies have shown that the gut microbiota yields significant effects on ICIs associated with microbiota composition/diversity, regulating efficacy and toxicity through metabolic and immune-mediated mechanisms (Routy et al., 2018; Hu et al., 2024). Gut microbiota composition profoundly impact the clinical response in melanoma patients undergoing immunotherapy, furthermore, fecal microbiota transplantation overcomes immunotherapy resistance (Davar et al., 2021; Gopalakrishnan et al., 2018; Szostak et al., 2024). Identifying the relationship between gut microbiota and tumor response to ICIs highlights that gut microbiota represents a potential biomarker and therapeutic target. The past decade has witnessed a burgeoning interest in harnessing probiotic to maintain a healthier gut microbiota and improve clinical outcomes of cancer therapy (Zheng et al., 2020; Tomita et al., 2020). Therefore, probiotic adjuvant therapy has gradually become a research hotspot to potentiate ICIs.

*Bacteroides fragilis* (BF839) is a gram-negative bacillus isolated from a healthy baby in September 1983, widely used as probiotic therapy in China to improve gut microbiota dysbiosis-related symptoms such as constipation and diarrhea (Geng et al., 2023). It has been confirmed that the specific response of T cells to *Bacteroides fragilis* was associated with the efficacy of CTLA-4 blockade (Vetizou et al., 2015). Accordingly, BF839 represents a potential therapeutic approach to improve the immunotherapy outcomes of cancer patients. The present study aimed to describe the relationship between BF839 and ICIs sensitivity. We performed 16S rRNA sequencing and RNA sequencing to investigate the mechanisms of BF839 in regulating antitumor immunity. Using B16-STING-KO mouse melanoma cells, we confirmed that BF839 inhibits tumor growth via cGAS-STING signaling. Our study emphasized that BF839 worked synergistically with anti-PD-1 antibody and induced tumor regression, mediated by increased CD8^+^T cell infiltration. Oral administration of probiotic BF839 greatly improved ICIs sensitivity in advanced solid tumors. These findings provide a strong theoretical basis for improving tumor response to ICIs by BF839 in clinical practice.

## 2 Methods

### 2.1 Cell lines

B16F10 (C57BL/6 mouse melanoma) cells and B16-STING-KO cells were kindly gifted by Dr. Xiaojun Xia at Sun Yat-Sen University Cancer Center. B16-STING-KO cells were constructed by stably knockout STING gene on B16F10 cells by CRISPR/Cas9 technology. All cell lines were maintained with RPMI 1640 (Invitrogen) containing 10% FBS and 1% penicillin–streptomycin at 37°C and 5% CO_2_.

### 2.2 *In vivo* treatments

Six-week-old female C57BL/6 mice were purchased from SPF (Beijing) Biotechnology and maintained under specific pathogen free (SPF) conditions. Each mouse was subcutaneously inoculated with melanoma cells (0.5 × 10^6^ cells/mouse). Nearly 1 week before the injection of melanoma cells, the mice were treated with BF839 (3.3 mL/kg, 5 × 10^6^CFU, ig, daily), HK-BF839 (hot kill with 60°C 2 h, 3.3 mL/kg, ig, daily), Bifico (42 mg/mouse, 5 × 10^6^CFU, ig, daily), respectively. When tumors reached an average volume of approximately 200mm^3^, mice received anti-PD-1 antibody intraperitoneal injection (100 μg/mouse, once every 3 days). We collected fecal samples from 3 mice (every 9 days during the treatment) and fed each mouse separately to reduce the chance of cross-contamination through faecophagy. The specific schemes of treatment plans were described in the Figures and legends. Tumor volume was calculated as follows: 0.5 × tumor length × (tumor width)^2^. Immunohistochemical staining of CD8^+^T (98941S, CST) cells and Foxp3^+^T (12653S, CST) cells was performed on mouse tumor tissues according to the instructions.

### 2.3 Patient

We retrospectively evaluated 29 patients with advanced solid tumors who received probiotic BF839 adjuvant therapy (20 mL, 1 × 10^8^CFU, po, daily, Totem, Dalian, China) combined with ICIs and chemotherapy at The Second Affiliated Hospital, Guangzhou Medical University (Guangzhou, Guangdong, China). Treatment was continued until unacceptable toxicity. BF839 adjuvant therapy duration longer than 4 months was defined as long-term treatment group, BF839 adjuvant therapy duration shorter than 4 months was defined as short-term treatment group. The survival time was verified in the patient’s follow-up records.

### 2.4 16S rRNA sequencing and analysis

Fresh fecal samples were collected and stored in liquid nitrogen. The DNA was extracted from fecal samples, after which the specific primers with a barcode were used to PCR amplification. The fragment length and concentration of PCR products were detected by 1% agarose gel electrophoresis. According to the concentration of PCR products, the volume of each sample was calculated, and the PCR products were mixed. Gel recovery kit was used to recover PCR mixed products and TE buffer to recover target DNA fragments. Finally, we used the Illumina Nova 6000 sequencing platform 250PE for sequencing. The original data obtained by sequencing were underwent quality control, filtering and splicing to obtain high-quality clean data. The final data for further species community analysis, diversity analysis and difference analysis. Sequencing and statistical analysis were performed by Magigene (Guangzhou, China).

### 2.5 RNA sequencing and bioinformatic analysis

Fresh tumor samples were collected and stored in liquid nitrogen. Total RNA was extracted using Trizol reagent kit (Invitrogen, Carlsbad, CA, United States). RNA quality was assessed on an Agilent 2100 Bioanalyzer (Agilent Technologies, Palo Alto, CA, United States) and checked using Rnase free agarose gel electrophoresis. After total RNA was extracted, eukaryotic mRNA was enriched by Oligo (dT) beads. Then the enriched mRNA was fragmented into short fragments using fragmentation buffer and reverse transcribed into cDNA with random primers. Second-strand cDNA was synthesized by DNA polymerase I, RNase H, dNTP and buffer. Then the cDNA fragments were purified with QiaQuick PCR extraction kit (Qiagen, Venlo, The Netherlands), end repaired, poly(A) added, and ligated to Illumina sequencing adapters. The ligation products were size selected by agarose gel electrophoresis, PCR amplified, and sequenced using Illumina novaseq 6000 by Gene Denovo Biotechnology Co (Guangzhou, China).

RNA differential expression analysis was performed by DESeq2 software between two different groups. The genes with the parameter of false discovery rate (FDR) below 0.05 and absolute fold change ≥ 2 were considered differentially expressed genes, after which GO and KEGG enrichment analyses were performed. We performed gene set enrichment analysis using software GSEA and MSigDB to identify whether a set of genes in specific pathways terms shows significant differences in two groups. Briefly, we input gene expression matrix and rank genes by SinaltoNoise normalization method. Enrichment scores and *p* value was calculated in default parameters.

### 2.6 Statistical analysis

Descriptive statistics was used to describe demographic data and clinical characteristics. Comparisons between two groups were analyzed using a two-tailed unpaired Student’s t test. Comparisons between multiple groups were analyzed using one-way ANOVA or two-way ANOVA for tumor growth study. For the difference analysis of alpha diversity between groups, parametric test and non-parametric test will be conducted, respectively. If there were only two groups, the Student’s t-test or wilcox rank sum test were used; if there were more than two groups, the Kruskal-Wallis rank sum test or one-way ANOVA were used. Bray-curtis algorithm was used to analyze the difference of beta diversity. A *p* value < 0.05 was considered statistically significant. * indicates *p* < 0.05, ** indicates *p* < 0.01, *** indicates *p* < 0.001, ns indicates *p* > 0.05, that is, no statistical difference. All data were analyzed using GraphPad Prism 7 (GraphPad Software).

## 3 Results

### 3.1 Patient characteristics

Twenty nine patients with advanced solid tumors at The Second Affiliated Hospital, Guangzhou Medical University (Guangzhou, Guangdong, China) were enrolled. The baseline clinical characteristics of patients were shown in Table 1. The patients were divided into long-term treatment group (15 patients; 51.72%) and short-term treatment group (14 patients; 48.28%). Fifteen patients (51.72%) were younger than 65 years old. Female patients comprised 13.78% of participants. The most common histopathology was lung cancer (15 patients; 51.72%), followed by liver cancer (7 patients; 24.14%). Twenty two patients (75.86%) were first-line treatment and 7 patients (24.14%) were second or more line treatment. The Eastern Cooperative Oncology Group (ECOG) performance status score was 0–1 in 28 patients (96.55%) and 2 in 1 patients (3.45%).

**Table 1:** Patient characteristics.

Characteristics	Long-term treatment	Short-term treatment
No. of patients	15	14
Age (years)		
<65	10	5
≥65	5	9
Gender		
Male	13	12
Female	2	2
ECOG score		
0–1	14	14
2	1	0
No. of prior therapies		
0	10	12
1 or more	5	2
Tumor type		
Lung cancer	7	8
Liver cancer	3	4
Rectal cancer	3	0
Ovarian cancer	1	0
Gastric cancer	0	1
Embryonal carcinoma	1	0
Duodenal carcinoma	0	1
Anti-PD-1 antibody		
Sintilimab	11	7
Triprolizumab	3	0
Pembrolizumab	1	3
Camrelizumab	0	3
Nivolumab	0	1

### 3.2 Long-term treatment of BF839 prolonged survival in immunotherapy patients

**Figure 1:**
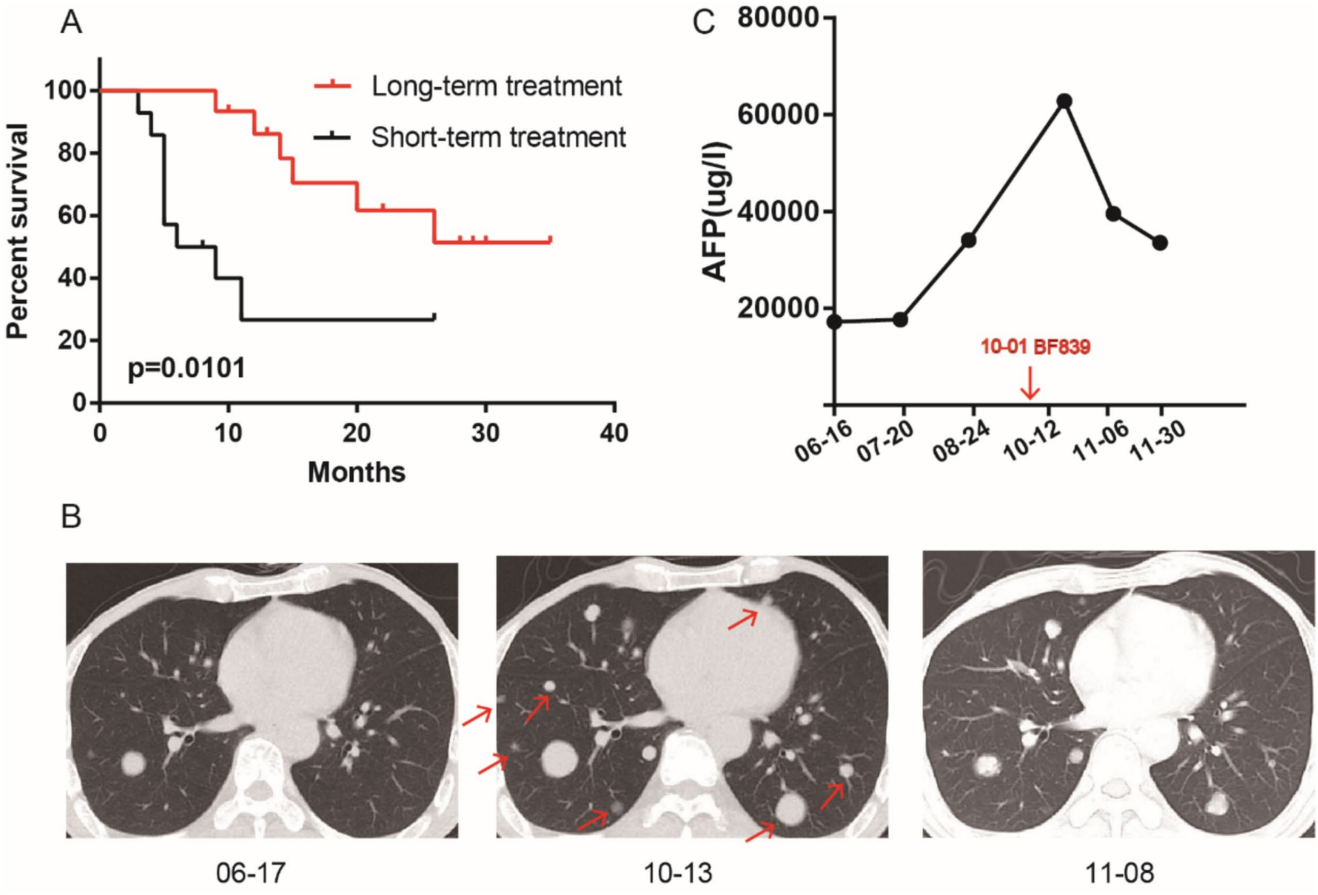
Long-term oral administration of BF839 improved the survival of patients who received ICIs. **(A)** Survival analysis of BF839 short-term treatment group and long-term treatment group. **(B)** The representative lung metastases pictures of BF839 pre-treatment and post-treatment. **(C)** The AFP level of BF839 pre-treatment and post-treatment.

Our previous study found that BF839 adjuvant therapy significantly reduce the adverse effects of chemotherapy in patients, but did not affect the efficacy of chemotherapy (Zeng et al., 2024). Based on this, we retrospectively investigated whether probiotic BF839 prolonged survival in patients undergoing ICIs and chemotherapy. These patients were divided into two groups based on the duration of BF839 treatment: long-term treatment group (>4 months) and short-term treatment group (<4 months). Compared with short-term treatment group, patients in long-term treatment group had longer overall survival (OS) (*p* = 0.0101, Figure 1A). The median OS in short-term treatment group was 7.5 months, but not observed in long-term treatment group. The 1-year survival rate in long-term treatment group was 86.15%, in short-term treatment group was 26.67%. The 2-year survival rate in long-term treatment group was 51.39%, in short-term treatment group was 26.67%. A patient with lung metastasis after liver cancer surgery received four cycles of chemotherapy combined with ICIs, and progressive disease was observed at each pre-treatment review (Figure 1B). The tumor marker AFP increased from 17,249 to 62,858 μg/mL. This patient was defined as primarily resistant to ICIs. On the basis of ICIs, oral BF839 treatment for 1 month, and CT review indicated partial response, with a decrease from 62,858 to 39,564 μg/mL observed for AFP (Figure 1C).

### 3.3 BF839 inhibited tumor growth by cGAS-STING

**Figure 2:**
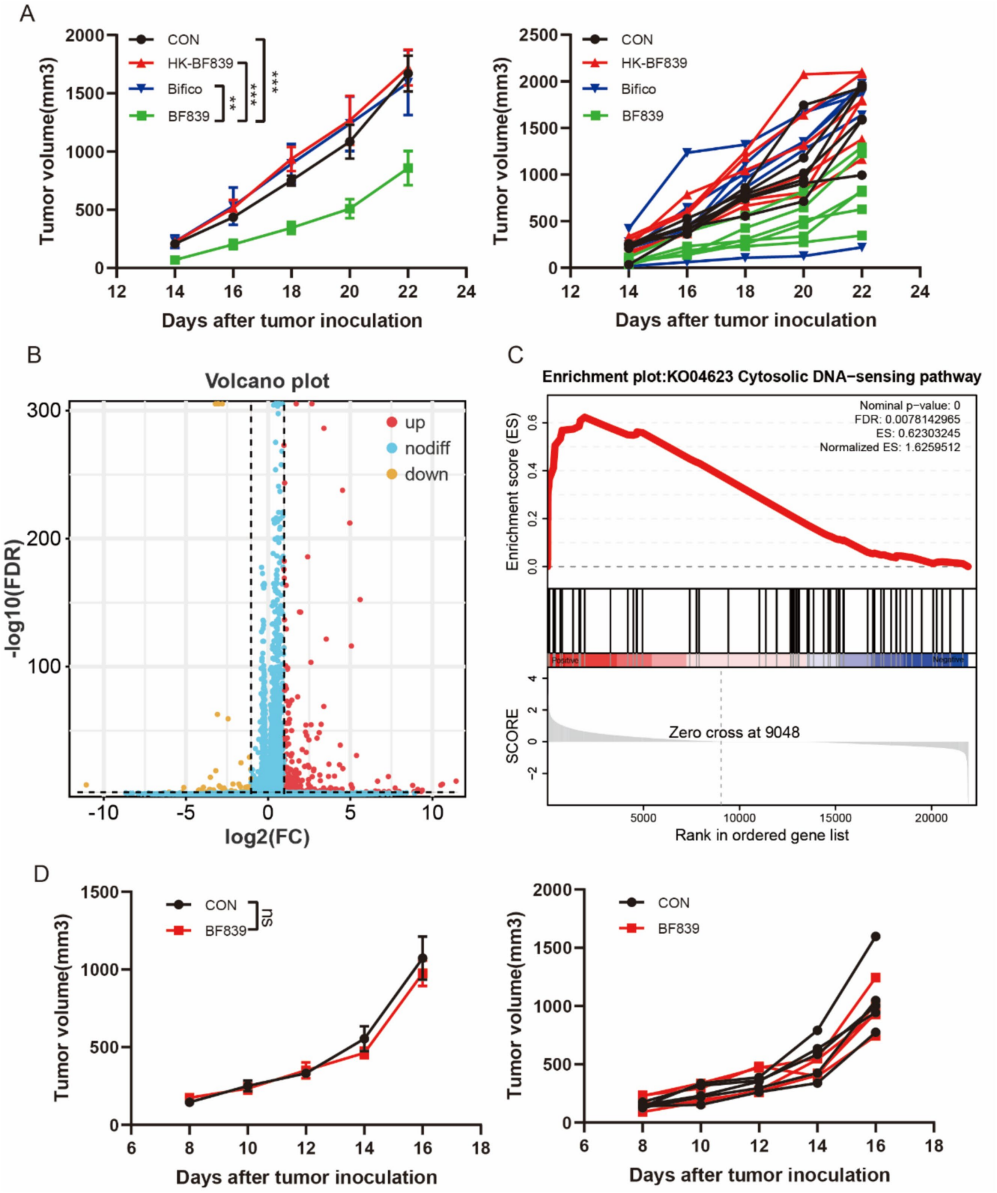
BF839 inhibited tumor growth through cGAS-STING pathway. **(A)** Tumor volumes of B16 tumor-bearing mice treated with PBS, BF839, HT-BF839, or Bifico, data are expressed as the mean ± SEM, *n* = 6 per group. **(B)** A total of 412 differentially expressed genes were identified by RNA-seq analysis in PBS and BF839 groups. **(C)** GSEA analysis of Cytosolic DNA sensing pathway. **(D)** Tumor volumes of B16-STING-KO tumor-bearing mice treated with PBS or BF839, data are expressed as the mean ± SEM, *n* = 6 per group.

We used a mouse melanoma cell (B16) to test the antitumor potential of BF839, HK-BF839 (hot kill), and Bifico (*Bifidobacterium,* a frequently used probiotic). We found that daily oral administration of BF839 starting 1 week before B16 tumor cell engraftment efficiently restrained tumor growth (Figure 2A). However, HK-BF839 did not inhibit tumor growth (Figure 2A). To explore the anti-tumor mechanism of BF839, we performed an RNA-seq analysis of tumor tissues (control group VS BF839 group). There were 347 genes with increased expression and 65 genes with decreased expression in the BF839 group (Figure 2B). The heatmap of all differential expressed genes was shown in Supplementary Figure S1. A pathway enrichment analysis of 412 differentially expressed genes revealed that Immune system process, Immune response, Defense response, Regulation of immune system process and Response to bacterium signaling pathway were significantly enriched (Supplementary Figure S2). Gene set enrichment analysis (GSEA) revealed that the Cytosolic DNA sensing (cGAS-STING) pathway was positively enriched in the BF839 group (Figure 2C). To investigate the role of cGAS-STING pathway in anti-tumor of BF839, we constructed B16-STING-KO cell model. We found that BF839 treatment did not inhibit B16-STING-KO tumor growth (Figure 2D). These results suggest that BF839 induced tumor suppression through cGAS-STING pathway.

### 3.4 BF839 promoted CD8^+^T cell infiltration and enhanced anti-PD-1 therapy efficacy

**Figure 3:**
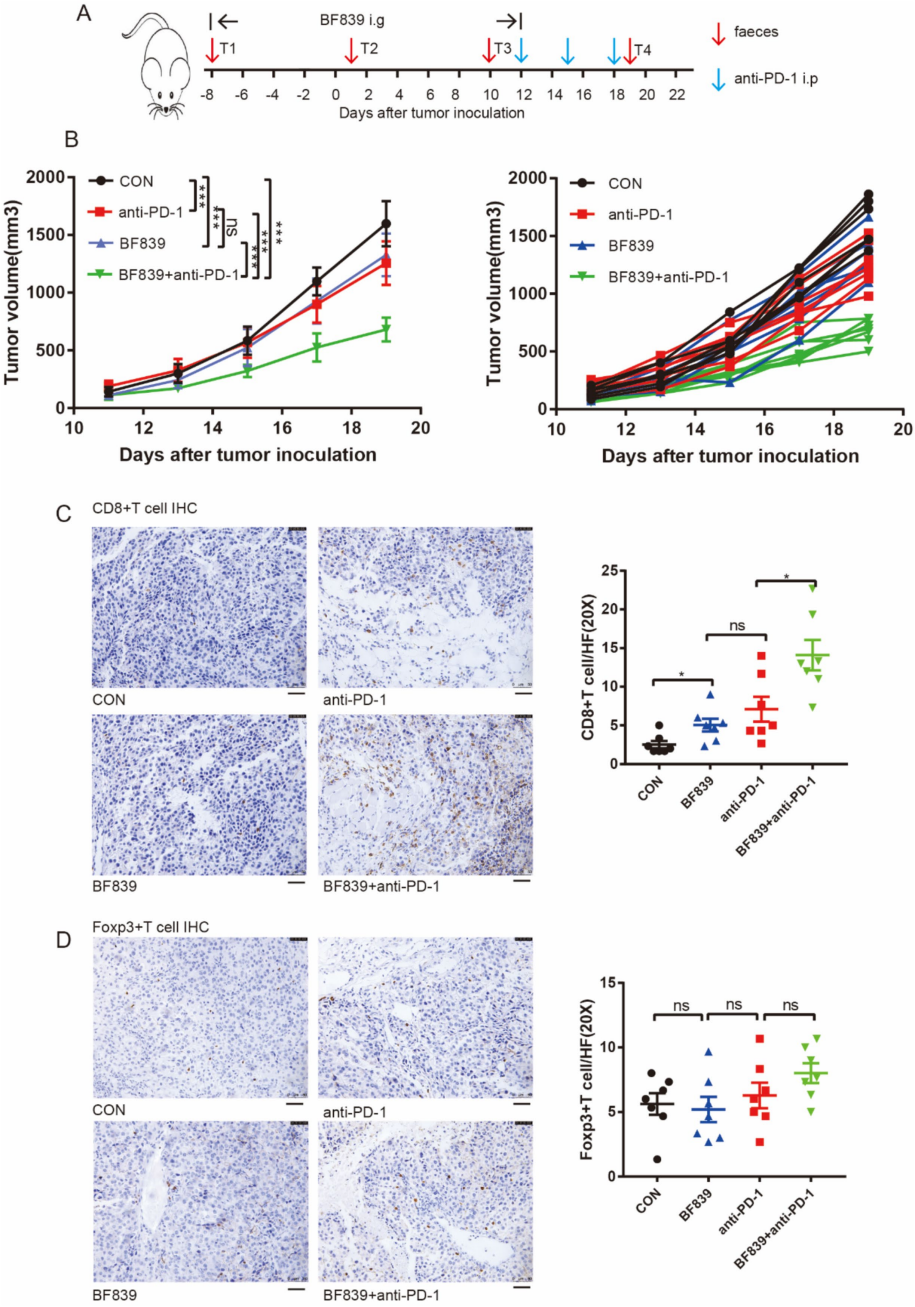
Combination of BF839 with anti-PD-1 promoted CD8^+^T cell infiltration. **(A)** Therapeutic plan of B16 tumor-bearing mice. **(B)** Tumor volumes of B16 tumor-bearing mice treated with PBS, BF839, anti-PD-1, or BF839 + anti-PD-1, data are expressed as the mean ± SEM, *n* = 7 per group. **(C)** Representative CD8^+^T cell IHC staining of the tumor tissue and quantitation of CD8^+^T cell, data are expressed as the mean ± SEM, *n* = 7 per group. Scale bars: 50 μm. **(D)** Representative Foxp3^+^T cell IHC staining of the tumor tissue and quantitation of Foxp3^+^T cell, data are expressed as the mean ± SEM, *n* = 7 per group. Scale bars: 50 μm.

We conducted experiments in a mouse melanoma model to investigate whether BF839 has synergistic efficacy with anti-PD-1 therapy. Briefly, the mice were randomly divided into four groups: control group, BF839 group, anti-PD-1 group, and BF839 + anti-PD-1 group. Nearly 1 week before the injection of B16 tumor cells, mice in the BF839 group and BF839 + anti-PD-1 group received daily oral administration of BF839. When tumors reached an average volume of approximately 200mm^3^, mice received anti-PD-1 antibody intraperitoneal injection. Mice fecal samples were collected every 9 days during the treatment course. A detailed overview of the treatment plan was shown in Figure 3A.

We found that monotherapy with either BF839 or anti-PD-1 antibody significantly inhibited tumor growth, while BF839 therapy improved the therapeutic efficacy of anti-PD-1 antibody (Figure 3B). To further verify the effect of BF839 treatment on tumor microenvironment, mouse tumor tissues were collected for immunohistochemical (IHC) staining. IHC staining showed that BF839 treatment promoted CD8^+^T cell infiltration in the tumor microenvironment, especially when combined with anti-PD-1 antibody treatment (Figure 3C). However, there was no significant effect on Foxp3^+^T cell infiltration (Figure 3D). Taken together, these results suggest that BF839 and anti-PD-1 antibody have synergistic efficacy and promote CD8^+^T cell infiltration.

### 3.5 BF839 treatment increased the diversity of gut microbiota

**Figure 4:**
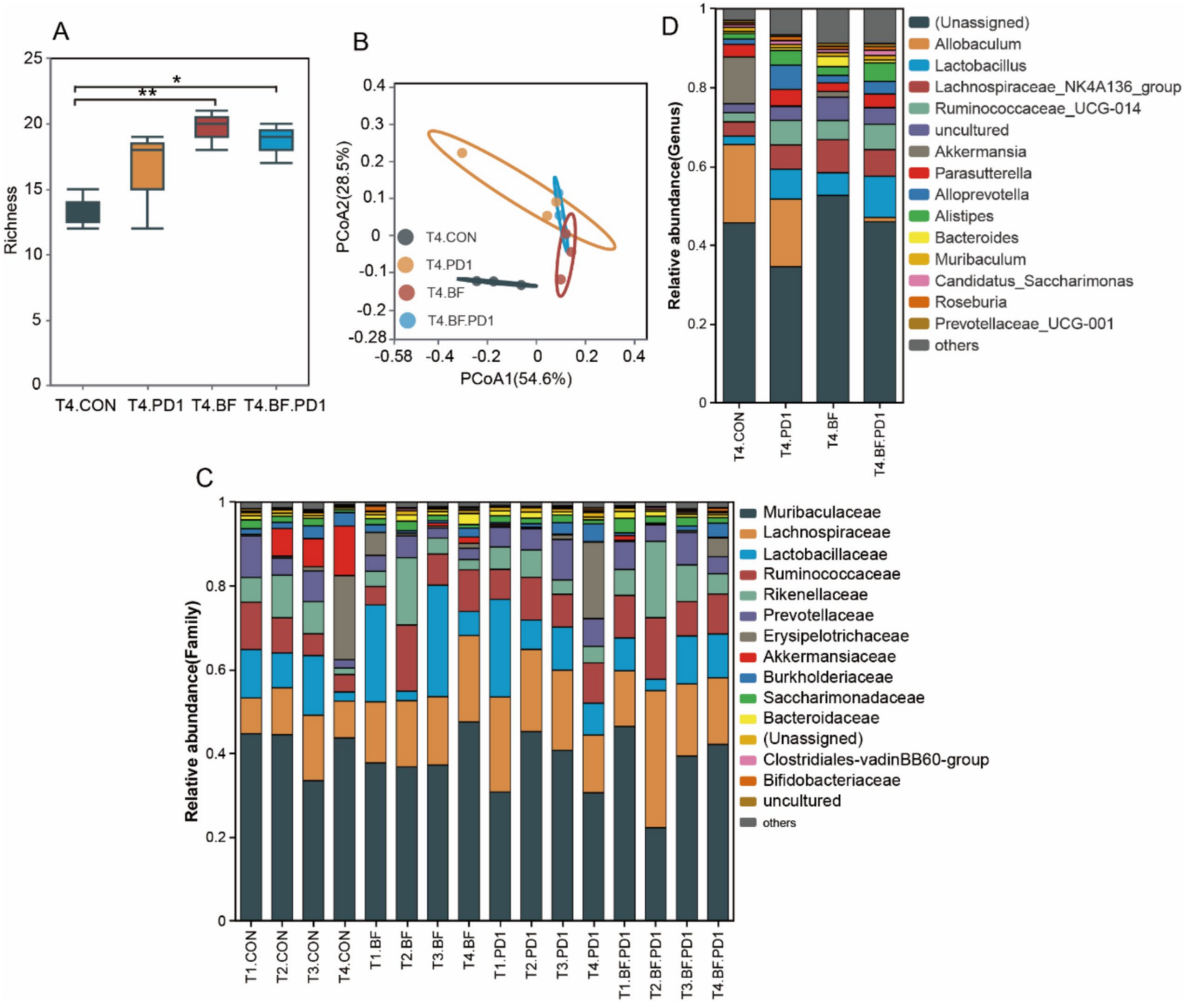
BF839 treatment increased the diversity of gut microbiota. **(A)** Gut microbiota diversity analysis at genus level. **(B)** Principal coordinates analysis (PCoA) at genus level. **(C)** Gut microbiotal community composition analysis of all samples at family level. **(D)** Gut microbiotal community composition analysis at genus level.

First, we performed 16 S rRNA sequencing to analyze the structure of gut microbiota in mice treated with BF839. The richness indice, which reflect gut microbiota diversity, was significantly lower in control group than in BF839 treatment group (Figure 4A). Although anti-PD-1 antibody treatment increased gut microbiota diversity compared with control group, the difference was not statistically significant (Figure 4A). We used principal coordinate analysis (PcoA) to profile the microbial space, showed a significant difference between control group and BF839 treatment group (Figure 4B). The distribution of T4.BF group samples were closer to T4.BF.PD1 group samples, suggesting greater similarity in the microbial community composition between the two groups (Figure 4B). In family level, *Muribaculaceae, Lachnospiraceae, Lactobacillaceae*, and *Ruminococcaceae* were the dominant families (Figure 4C). Compared with non-BF839 treatment group, the level of *Allobaculum* was lower in BF839 treatment group in genus level (Figure 4D). These results demonstrate that BF839 therapy improve gut microbiota diversity.

## 4 Discussion

There is considerable evidence that the probiotics affects anti-tumor immunity in solid tumors (Li et al., 2024; Liu et al., 2024; Roy and Singh, 2024). In our study, the preclinical experiments showed promising activity: BF839 combined with anti-PD-1 significantly inhibited tumor growth and promoted CD8^+^T cell infiltration in the tumor microenvironment. Mice fecal samples 16S rRNA sequencing showed that gut microbiota diversity was significantly lower in non-BF839 treatment group than in BF839 treatment group. Mice tumor samples RNA-seq analysis revealed that the cGAS-STING pathway was positively enriched in the BF839 group. Furthermore, this study revealed the potential synergistic effect of probiotic BF839 on ICIs efficacy in patients with advanced solid tumors. Compared with BF839 short-term treatment group, patients in BF839 long-term treatment group had longer OS. The 1-year survival rate in long-term treatment group was 86.15%, in short-term treatment group was 26.67%. The 2-year survival rate in long-term treatment group was 51.39%, in short-term treatment group was 26.67%. These findings provide a strong theoretical basis for the clinical application of BF839 to improve tumor response to ICIs.

Probiotics have been reported to benefit human health, the immune system, inflammation and digestive tract diseases strongly affect the gut microbiota (Sehrawat et al., 2021). Given that probiotics treatment improve the imbalance and diversity of gut microbiota, it has a positive effect on tumor immunotherapy (Derosa and Zitvogel, 2022; Zitvogel et al., 2022). A variety of probiotics have been identified to improve immunotherapy. It has been documented that the probiotic *Clostridium butyricum* enhanced the efficacy of ICIs in patients with NSCLC (Tomita et al., 2020). CBM588 is a bifidogenic live bacterial product that has been reported to improve clinical outcomes in patients with metastatic renal cell carcinoma treated with nivolumab and ipilimumab; the PFS was significantly longer in patients receive nivolumab and ipilimumab with CBM588 than without (12.7 months versus 2.5 months) (Dizman et al., 2022). In another study, administering *Bacteroides fragilis* by oral gavage increased gut microbiota diversity and beneficial commensal bacteria, significantly alleviating acute and chronic GVHD development (Sofi et al., 2021).

The gut microbiota can be manipulated through several strategies, including fecal microbiome transplantation, a safe and effective treatment for recurrent *Clostridium difficile*, that is even used in cancer treatment (van Nood et al., 2013; McQuade et al., 2019). In a study of melanoma patients, 6 of 15 immunotherapy-refractory patients who received fecal microbiota transplantation from immunotherapy responders, were re-sensitized to immunotherapy (Davar et al., 2021). Another approach is bacterial genetic engineering technology, it is possible to improve anti-tumor response to ICIs by modifying gut microbiota or metabolites (Ma et al., 2023). To date, *Escherichia coli, Bifidobacterium, Listeria*, and *Salmonella* engineered by gene attenuated, nutrient deficient, and inducible have shown anti-tumor effects in preclinical models (Chang et al., 2020; Taniguchi et al., 2016; Liang et al., 2019; Zhou et al., 2018; Lee et al., 2021). Intratumoral injection of *Escherichia coli* Nissle 1917 increased intracellular L-arginine concentration, induced CD4^+^ T cell and CD8^+^ T cell infiltration, and produced synergistic antitumor effect when combined with anti-PD-L1 (Canale et al., 2021). Additionally, adjusting diet and lifestyle have been reported to participate in the regulation of gut microbiota (Wolter et al., 2021; Simpson et al., 2022; Spencer et al., 2021). Calorie restriction without malnutrition reduce the risk of cancer development, also lead to gut microbiota remodeling (Mao et al., 2023). Mechanically, calorie restriction induced anti-tumor effect dependent on acetate production and the accumulation of interferon-γ^+^CD8^+^ T cells. In a latest study, vitamin D levels were observed to affect gut microbiota composition in favor of *Bacteroides fragilis*, which in turn improved cancer antitumor immunity (Giampazolias et al., 2024). Our study demonstrated that combining BF839 with anti-PD-1 not only increased functional CD8^+^ T cells infiltration but also enhanced anti-PD-1 therapy efficacy. RNA-seq analysis revealed the Cytosolic DNA sensing (cGAS-STING) pathway was positively enriched in the BF839 group.

Biomarkers such as PD-L1 expression and tumor mutation burden were associated with ICIs efficacy. Recently, gut microbiota diversity has been studied as one of the variables for ICIs response heterogeneity (Zhang et al., 2021). There is an increasing consensus that antibiotics affect the gut microbiota, including the loss of specific microbiota and poor diversity (Rashidi et al., 2021). Gut microbiota disruption caused by antibiotic use impairs the efficacy of chemotherapy and immunotherapy (Erttmann et al., 2022; He et al., 2021). Among cancer patients treated with ICIs, antibiotic use was negatively associated with PFS and OS, suggesting that a harmonious gut microbiota diversity is required for effective antitumor effect (Derosa et al., 2018; Cheung et al., 2021). Using probiotics to modulate antibiotic-associated dysbiosis and gut microbiota diversity represents a potential strategy to improve ICIs treatment (Routy et al., 2018). In our study, mice fecal samples 16S rRNA sequencing showed that gut microbiota diversity was significantly lower in non-BF839 treatment group than in BF839 treatment group. Oral administration of BF839 improved the response to ICIs in patients with advanced solid tumors, especially in the long-term treatment group. It is highly conceivable that short-term oral BF839 does not form effective probiotic colonization. The optimal duration of BF839 treatment remains to be determined. BF839 is widely used as probiotic therapy in China to improve gut microbiota dysbiosis related symptoms such as constipation and diarrhea. Therefore, BF839 has the potential to ameliorate gut microbiota disorders during immunotherapy.

There are some limitations in this study. Firstly, all participants were enrolled from the single center, a multicenter cohort should be conducted in the future to further strengthen these results. Genetic background, dietary habits, comorbidities and other factors affect the diversity of gut microbiota (Lozupone et al., 2012; David et al., 2014; Wang et al., 2024). Secondly, due to the retrospective nature of this study, we were unable to obtain fecal samples and tumor biopsy specimens from the patients for the analysis of probiotic colonization and the tumor microenvironment. Finally, in the mouse model, the STING pathway involved in the promotion of anti-tumor immunity by BF839, and its molecular mechanism needs further research.

In conclusion, our data suggest that probiotic BF839 induced tumor suppression was regulated by the cGAS-STING pathway. BF839 enhanced ICIs sensitivity by improving gut microbiota diversity and promoting the accumulation of CD8^+^T cell. Our study demonstrate the therapeutic promise of modulating the gut microbiome in tumor patients treated with ICIs and provides a new strategy to enhance the efficacy of ICIs.

## Data Availability

Sequence data generated in this study was uploaded to the Baidu Netdisk. It can be accessed via https://pan.baidu.com/s/1lpMuHppiYPxNE_nO0XXkWw with the accession number bf83. The data that support the findings of this study are available from the corresponding author upon reasonable request.

## Supplementary material

Figure S1 Heatmap of 412 differentially expressed genes.

Figure S2 GO analysis of differentially expressed genes (FC > 2 or FC < 0.5 and *p* value < 0.05) in PBS and BF839 groups.
