# Supplementary figures and images for: The therapeutic promise of probiotic Bacteroides fragilis (BF839) in cancer immunotherapy
Source: Front Microbiol. 2025 Mar 31;16:1523754. doi: 10.3389/fmicb.2025.1523754 (PMC11995047; doi:10.3389/fmicb.2025.1523754)

heatmap

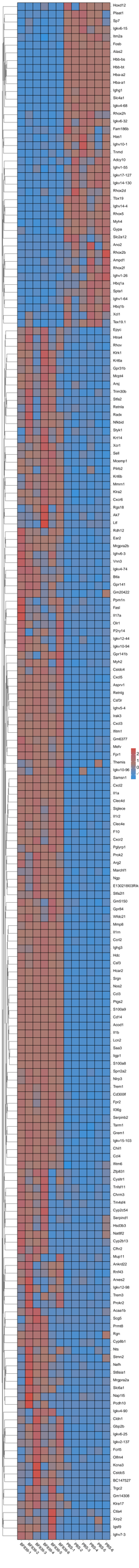

Supplement: Figure S1 — Heatmap of 412 differentially expressed genes. [file Data_Sheet_1.PDF]

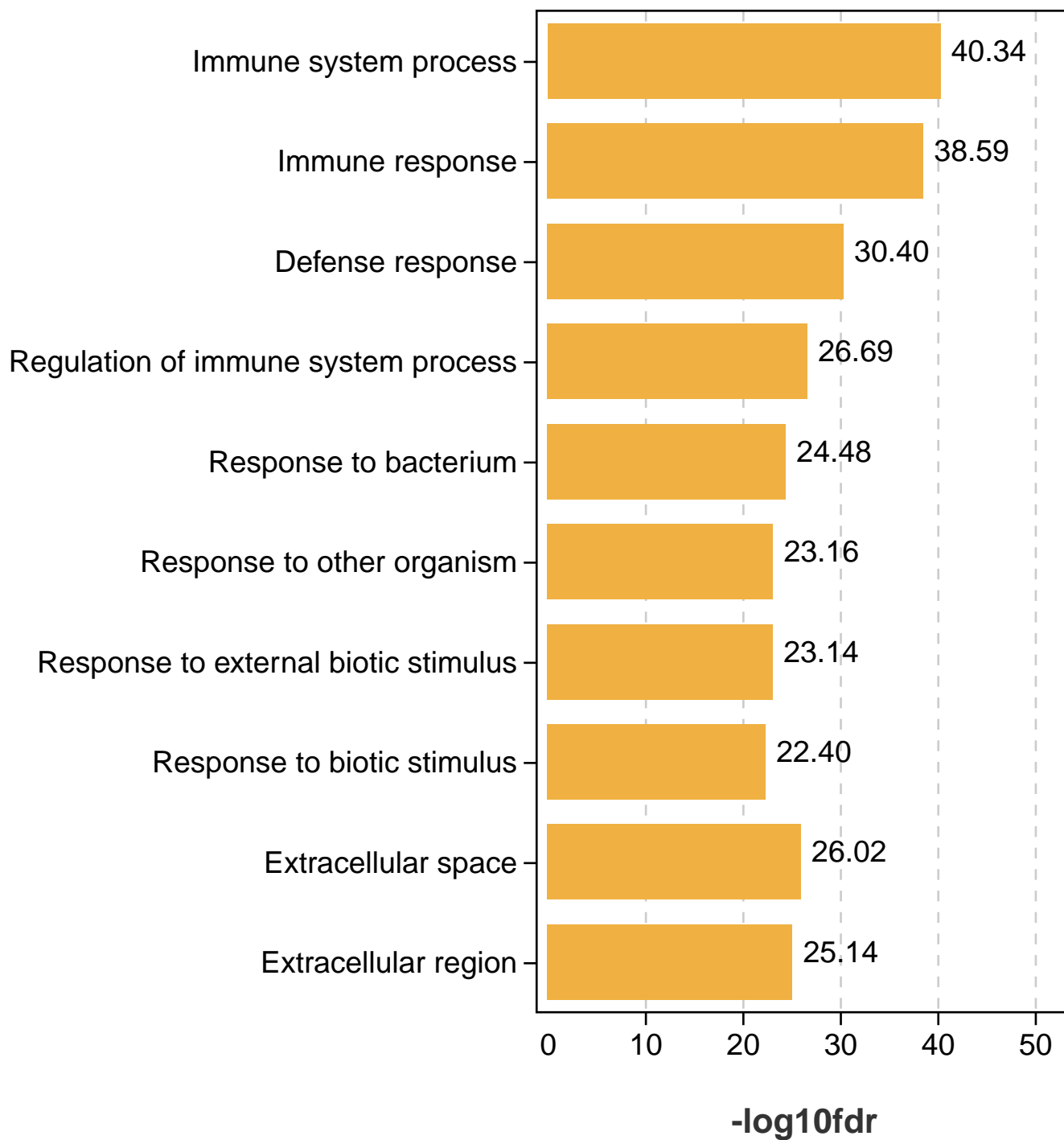

Supplement: Figure S2 — GO analysis of differentially expressed genes (FC > 2 or FC < 0.5 and p value < 0.05) in PBS and BF839 groups. [file Data_Sheet_2.PDF]
